# Supplementary material for: Consumption of sorghum-based products and their impact on blood glucose and satiety in adult patients: A protocol for systematic review and meta-analysis
Source: PLoS One. 2025 Sep 4;20(9):e0330998. doi: 10.1371/journal.pone.0330998 (PMC12410867; doi:10.1371/journal.pone.0330998)
Supplement: Attachment — Submitted filename: Response to Reviewers PLOS ONE.pdf [file pone.0330998.s002.pdf]

Response to Reviewers

PLOS ONE Decision: Revision required [PONE-D-24-05305]

Vitória, ES, Brazil, March 22th, 2025

Dear Federico Vita,
Academic Editor.

We sincerely thank the editors and reviewers for their valuable comments and suggestions regarding the structure and content of our manuscript. We have carefully revised the manuscript in response to each point raised, and a detailed description of the changes made is provided below, item by item.

We are grateful for the thoughtful and constructive feedback, which has significantly contributed to improving the quality and clarity of our work. We fully agree with all the comments and have implemented the recommended revisions throughout the manuscript.

Below, we provide detailed responses to each of the editor's comments.

1) The article has finally been revised. As you can see, each reviewer raised a series of issues that must be addressed before resubmitting the revised version. These issues include the need to improve the reference section related to current and past year, the need to clarify some points in both the discussion and the material and methods section, and an extensive text revision by a native English speaker.

Response: The References, Materials and Methods, and Discussion sections have been thoroughly revised. Additionally, the entire manuscript has been carefully reworded for grammatical accuracy and clarity by a native English speaker. All modifications are highlighted in track-changes.

Reviewer #1: Major revision

The authors present a systematic review protocol to identify and evaluate the evidence of the effectiveness of sorghum used in foods on blood glucose levels as well as satiety in adult patients.The introduction is long and contains unnecessary text. The suggestion is that the authors remove the entire paragraph between lines 96 and 106.

Response: The suggestion has been accepted, and the text has been excluded.

Furthermore, they should remove the excerpt "Therefore, this study aims to identify and evaluate the evidence of the effectiveness of sorghum used in foods on blood glucose levels as well as satiety in adult patients." at the end of the last paragraph, as this objective was mentioned previously.

Response: The suggestion has been accepted, and the text has been excluded.

In the methodology, the authors need to clarify how the studies to be included evaluated the primary outcomes "Impact on blood glucose levels and satiety", as this term is very vague. What will be assessed regarding the glycemic profile? Fasting blood glucose, glycated hemoglobin, HOMA index? How is the impact on satiety assessed? Are there instruments validated in clinical trials, which ones?

Response: The suggestion has been accepted and incorporated into the revised manuscript.

Furthermore, should they elucidate how the secondary outcome of overweight and obesity should be assessed? Body mass index, BMI, follows international standards?

Response: The suggestion has been accepted and incorporated into the revised manuscript.

Still in this section, the authors need to include the subtopics Certainty of Evidence, explaining the use of GRADE for this assessment and Subgroup Analysis and Sensitivity, clarifying, if there is a meta-analysis, which subgroups

will be considered (Gender? Age group? Adults without and with diabetes?) and how they intend to carry out the sensitivity analysis.

Response: The suggestion has been accepted and incorporated into the revised manuscript.

Finally, they also needed to mention what the publication bias analysis will be like, Funnel Plot? Egger test?

Response: The suggestion has been accepted and incorporated into the revised manuscript.

In the discussion, they should add a paragraph in which they defend the strengths of the future systematic review and possible limitations such as the scarcity of studies with high quality and certainty of evidence, heterogeneity between studies, and small samples that may compromise the results and the certainty of the evidence.

Response: The suggestion has been accepted and incorporated into the revised manuscript.

"This future systematic review presents several strengths that enhance its relevance and methodological rigor. It will be the first to focus specifically on the effects of sorghum-based food consumption on blood glucose regulation and satiety in adults, addressing a critical gap in the current literature. The protocol adheres to established methodological standards, including the PRISMA-P checklist, and employs validated tools such as RoB 2 and ROBINS-I for risk of bias assessment. The comprehensive and unrestricted search strategy, as well as the inclusion of both randomized and non-randomized trials, further strengthens the review's breadth and potential impact. However, several limitations are anticipated. These include the potential scarcity of high-quality studies with strong methodological designs, small sample sizes, and heterogeneity among studies in terms of populations, interventions, and outcome measures. Such factors may compromise the robustness of the findings and reduce the overall certainty of the evidence. Despite these challenges, the systematic review will aim to address these issues through rigorous data synthesis, transparent reporting, and the application of the GRADE approach to evaluate the certainty of the evidence, ultimately providing a balanced and critical appraisal of the available research."

<u>Reviewer #2: Accept</u>

In this systematic review protocol with meta-analysis, the authors address the effectiveness of sorghum present in cereal-based foods on blood glucose levels and satiety in adult patients. This is a relevant topic as the consumption of these types of foods is often associated with a source of prevention for Diabetes Mellitus, Obesity and other non-communicable diseases.

My general impression is that the article is well written, proposing an approach with high method accuracy, especially in the database search strategy and in terms of assessing the risk of bias. This is an innovative theme, as it seeks evidence in clinical trials of the association between the consumption of a food and one of the most prevalent diseases in the world and costly for health systems.

The manuscript was structured following the recommendations of the PRISMA-P checklist. The search strategy is clear and well defined, using database subject indexers, Keywords and synonyms combined by Boolean operators. The authors selected the main databases to carry out the searches. They also specified planning for the use of tools to assess risks of bias in accordance with the Cochrane Handbook, such as, RoB2 and ROBINS-I.

Response: We sincerely appreciate your positive feedback and thoughtful evaluation of our manuscript.

Reviewer #3: Major comments

1. You need to describe appropriately your study population. In some cases, you refer to “adult patients” and in others to just “adults”.

In the eligibility criteria section, add more information on the health status of the studies’ participants that is accepted. For example, are you going to include studies in healthy volunteers? Are there any health conditions you will exclude e.g. gestational diabetes mellitus, malignancy, etc.? You should provide a detailed description.

- ✓ The suggestion has been accepted and incorporated into the text.

2. In the eligibility criteria section, you should refer to all the other types of studies that will be excluded e.g. animal studies, studies in individuals aged <18 years old, meta-analyses, conference papers, etc.

- ✓ The suggestion has been accepted and incorporated.

3. In the methods section refer to the duration of the studies you will use. For example, will you use acute studies, long-term studies, or both?

- ✓ The suggestion has been accepted and incorporated.

4. Replace “experimental studies” with “randomized controlled trials and non-randomized controlled trials” in the whole text.

- ✓ The suggestion has been accepted and incorporated.

5. What measures of satiety do you intend to use in your results? Visual analog scales or hormone levels?

- ✓ We intend to use both visual analog scales (VAS) and hormone levels as measures of satiety in our results. Our literature search has revealed that studies on satiety related to sorghum-based foods have employed a

variety of methodologies, including both subjective (VAS) and objective (hormone levels) measures. By incorporating both approaches, we aim to provide a comprehensive understanding of the impact of sorghum on satiety and to enhance the robustness of our findings.

6. In your search strategy, won't you refer to specific glycemic indices such as glycated hemoglobin A1c? What glycemic outcomes do you intend to use?

Response: We appreciate the reviewer's thoughtful question regarding the specification of glycemic indices in the search strategy. Given the potential for a limited number of studies that meet our inclusion criteria, we have opted to avoid overly specific terms in the search strategy in order to maximize the retrieval of relevant articles. Including particular glycemic indices—such as glycated hemoglobin A1c (HbA1c)—at the search stage might inadvertently exclude studies that assess blood glucose through alternative, yet equally valid, measures. Instead, we have incorporated these specific glycemic outcomes into the eligibility criteria. This approach provides greater flexibility during the search process while ensuring that the included studies are relevant to our primary research question. Specifically, we will consider studies that assess blood glucose using validated measures such as fasting blood glucose, HbA1c, and HOMA-IR. This strategy allows us to capture a broader and more comprehensive set of studies without compromising the specificity of our review objectives.

7. In the "Introduction" you should analyze the existing systematic reviews to show your difference (Lines 123-124); what they have examined.

Response: The suggestion has been accepted and incorporated into the revised manuscript.

Minor comments

1. Line 50: “response” should be replaced with “responses”

2. Lines 79-80: “Diabetes mellitus type 2 (T2DM)” should be replaced with “type 2 diabetes mellitus (T2DM)”
3. Line 80: a comma (,) is needed after “weight management”

4. Line 92: “BMI” need to be whole-written first

5. Line 95: You should use only lowercase for “Diabetes Mellitus”

6. Line 98: You should replace “diabetes” with either “DM” or “T2DM” as mentioned above in your manuscript to be consistent in your writing

7. Lines 98-99: “type 2” should be replaced with “T2DM”

8. Lines 99-100: “and is highly prevalent among the two main subtypes of DM” is mentioned again in the previous line, so it should be deleted

9. Lines 100-101: “This disease…choices”. Add reference to this sentence

10. Line 113: “dietary” should be deleted

11. Line 114: “glycemic index and load” should be mentioned abbreviated

12. Line 117: Add “with” after along

13. Lines 121-122: The comma (,) after such as is not needed. Replace with “enhancing” and “preventing”

14. Libe 127: The word “concentrates” is not appropriate for the meaning of your sentence. Moreover, replace “addresses” with “address”.

15. Line 136: “type 2 diabetes” should be replaced with “T2DM”

16. Line 142: You should delete “Moreover”

17. Line 152: “the scrutinize” should be deleted

18. Line 157: RCT and NRCT should be first whole-written and then abbreviated

Response: All minor comments have been fully addressed and corrected as requested. We thank the reviewer for the careful and constructive review.

<u>Reviewer #4: Major revision</u>

December 2023 is too old data. The authors need to add all papers in their filed up to today for this study to be current and in line with recent findings. When the authors do this work, I will be happy to review it. Until then, I do not agree to its publication.

Response: We appreciate the reviewer’s comment and fully agree with the importance of including the most up-to-date evidence to ensure the relevance and scientific rigor of this study. Accordingly, we confirm that the search strategy will cover all records from the inception of each database through February 2025, thereby incorporating the most recent and relevant publications. We thank the reviewer for this valuable observation and for their willingness to reassess the manuscript once the update is completed.

Best regards, The authors
