# Supplementary material for: Consumption of sorghum-based products and their impact on blood glucose and satiety in adult patients: A protocol for systematic review and meta-analysis
Source: PLoS One. 2025 Sep 4;20(9):e0330998. doi: 10.1371/journal.pone.0330998 (PMC12410867; doi:10.1371/journal.pone.0330998)
Supplement: Attachment — Submitted filename: Response to Reviewer May 28.docx [file pone.0330998.s003.docx]

**Response to Reviewers**

PLOS ONE Decision: Revision required [PONE-D-25-15492]

Vitória, ES, Brazil, May 28^th^, 2025

Dear Federico Vita,

Academic Editor.

We sincerely thank the editors and reviewers for their valuable comments and suggestions regarding the structure and content of our manuscript. We have carefully addressed each point raised and provide a detailed, item-by-item description of the changes made below.

We are grateful for the thoughtful and constructive feedback, which has greatly enhanced the quality and clarity of our work. We fully agree with all the comments and have incorporated the recommended revisions throughout the manuscript.

Below, we provide detailed responses to each of the editor’s comments.

**Reviewer #1: Major Revision**

Interesting study. Sound methodology. I agree with the authors that a lack of published material may limit the outcome, but at least this provides a sound basis for further empiric study.

**Response:** We sincerely appreciate your positive feedback and thoughtful evaluation of our manuscript.

**Minor points:**

1. Reframe your research question as 2 separate ones. Blood glucose and satiety will probably be linked, but not necessarily.

In addition, why include the word 'patients' in your research question? Shouldn't you include well individuals? If you just want participants who are ill, then specify the illness (e.g. Type 2 DM).

I would suggest revision to the following: "What is the effectiveness of incorporating sorghum into the diet on (a) blood glucose levels and (b) satiety in adults?”

**Response:**  We thank the reviewer for the thoughtful and constructive feedback. In response to the suggestion, we have revised the research question to clearly separate the outcomes and to use terminology that more accurately reflects the study population. The updated version has been incorporated into the manuscript accordingly.

2. P5, line 158, the sentence "In line with PICOS strategy, our research question is: " is not completed (but is answered before this in the paragraph)

**Response:** We appreciate your careful reading of our manuscript. The excerpt in question has been removed from the text.

**Reviewer #2**:

This is a systematic review protocol paper outlining the strategy for a future systematic review project on the role of sorghum on satiety and blood glucose. I note that this is a revision and I was not one of the original reviewers. My own reading of the paper is that it is acceptable for publication based on its ability to meet the methodological requirements of a planned protocol for a systematic review. The Introduction gives a satisfactory background justifying the study. With respect to the previous reviews, the authors have made the requisite corrections and have replied to the complaint of the last reviewer.

**Response:** We sincerely appreciate your thoughtful evaluation and positive feedback on our manuscript.

**Reviewer #3: Suggestions for Improvement**

1. Eligibility Criteria: Please provide the thought processes in the creation of the inclusion and exclusion criteria. Specifically, clarify the rationale for excluding older adults (>65 years) with diabetes/chronic diseases. Provide literature support for this decision, as this population may benefit from sorghum-based interventions.

**Response:** We are truly grateful for your thoughtful evaluation of our manuscript. This was indeed an oversight, and we sincerely appreciate you bringing it to our attention. Older adults are not excluded from our review and are, in fact, included in the search strategy, as this population - particularly those with chronic conditions - may benefit from sorghum-based dietary interventions. The excerpt suggesting their exclusion was removed to reflect our eligibility criteria accurately.

*Now reads, line 173: “Exclusion criteria: children, adolescents, and adults (age > 18 years of any ethnicity, diagnosed with diabetes mellitus or any other chronic disease).”*

2. Data Availability and Reproducibility: While the protocol states data will be made available upon completion, specifying a repository (e.g., GitHub, OSF) for extracted data and analysis code would align with best practices for open science. This is particularly relevant since the data are derived from publicly available studies.

**Response:**  We sincerely thank the reviewer for raising this important point regarding data sharing and reproducibility. In response, we have now specified the repository where the data and analysis code will be made available upon completion of the study. Specifically, we will use the Open Science Framework (OSF), a platform widely adopted in open science practices.

Accordingly, we have revised the “Data Availability” statement in the manuscript to read as follows:

“Upon completion of the review, all extracted data and analysis code will be made publicly available in the Open Science Framework (OSF) repository. The direct link to the repository will be provided in the final publication.”

We trust this modification addresses the reviewer’s concerns and strengthens the transparency and reproducibility of our study.

3. Active Voice Revision: The manuscript occasionally uses passive voice (e.g., Line 128, p. 4). Revising to active voice (e.g., *"This study employs systematic review methods to synthesize evidence..."*) would improve clarity, as recommended by the [CDC’s writing guidelines] (<https://www.cdc.gov/pcd/issues/2018/18_0085.htm>).

**Response:** The suggestion has been accepted and incorporated into the revised manuscript.
*Now reads, line 129: “This study employs systematic review methods to synthesize evidence, offering valuable insights into the effectiveness of sorghum-based dietary interventions for improving glycemic control and promoting satiety in adults.”*

4. Pilot Testing of Search Strategy: Consider running a trial search to ensure inclusion criteria are not overly restrictive, especially if the authors aim to capture key studies (e.g., prior reviews or known manuscripts).

**Response:** We appreciate the suggestion. A pilot testing of the search strategy was conducted during the initial stages of the protocol to ensure the inclusion criteria were appropriate and not overly restrictive. This preliminary test helped confirm the strategy’s ability to retrieve key studies and manuscripts in the field.

5. Optional Recommendations:

1. Publication of Analysis Code: Sharing statistical code (e.g., R/Python scripts for meta-analysis) as a supplement or in a repository would enhance reproducibility.

**Response:** We fully agree with the reviewer that sharing the statistical code is essential for ensuring reproducibility and transparency. As noted, all analysis scripts used for data synthesis (e.g., SPSS syntax files, R/Python scripts, or any additional computational tools applied) will be uploaded to the Open Science Framework (OSF) repository alongside the dataset upon completion of the review.

To reflect this commitment, we have further revised the "Data Availability" statement to explicitly mention the inclusion of statistical code. The revised version now reads:

“Upon completion of the review, all extracted data and statistical analysis code (e.g., SPSS, R, or Python scripts) will be made publicly available in the Open Science Framework (OSF) repository. The direct link to the repository will be provided in the final publication.”

We hope this revision adequately addresses the reviewer’s valuable suggestion and demonstrates our commitment to open science practices.

**Overall Recommendation**

This protocol is well-conceived and merits publication after addressing the minor revisions above. The study has the potential to inform dietary guidelines and future research on sorghum’s health benefits. I commend the authors for their thorough approach and look forward to the completed review.

**Response:** We are truly grateful for your thoughtful evaluation and constructive feedback, which have greatly contributed to the improvement of our manuscript.

**Reviewer #4: Major comments**

In general, this seems to be a reasonable protocol for a systematic review of the topic chosen by the authors. My main concern is the lack of publications on this topic present in PubMed. If the majority of the analysis will be reliant on reports in clinical trials databases and secondary sources that are not peer-reviewed, this will result in a review with weak conclusions. It would make sense to at least identify additional appropriate conference abstract databases that could be included in the analysis, e.g. International Diabetes Federation, American Diabetes Association, etc.

**Response:**

We thank the reviewer for this thoughtful and constructive comment. We acknowledge that the current body of peer-reviewed literature on this specific topic may be limited. To mitigate this, and in line with the reviewer’s excellent suggestion, we have revised our protocol to include searches in the abstract repositories of major scientific organizations relevant to the scope of our study.

In particular, we have now added the following sources to our search strategy:

- **International Diabetes Federation (IDF) Congress Abstract Archive**
- **American Diabetes Association (ADA) Scientific Sessions Abstract Database**

These databases will be searched systematically to identify any additional unpublished or recent studies, abstracts, or relevant data that may not yet be indexed in PubMed or other traditional databases. This amendment aims to improve the comprehensiveness and inclusiveness of our evidence base and reduce the risk of publication bias.

We have also updated the "Search Strategy" section of the manuscript to reflect these additions.

**Specific points about manuscript:**

Search term #1 appears to be at odds with the exclusion criteria – why are you searching for “aged” subjects when these are excluded from the review?

**Response:**

We thank the reviewer for this pertinent and helpful observation. To clarify, older adults are not excluded from our review. The inclusion of the term “aged” in the search strategy is therefore consistent with our eligibility criteria, which encompass adults aged 18 years and older, regardless of upper age limit.

We have reviewed the manuscript and revised any wording that may have implied the exclusion of older adults in order to ensure internal consistency throughout the text.

We are grateful to the reviewer for highlighting this important point, which has contributed to improving the clarity and methodological transparency of our protocol.

Search term #6 should include the variant spelling “glycaemic” as this spelling was widely used until recently.

**Response:** The suggestion has been accepted, and the text has been incorpored.

- There is no evidence anywhere that the authors will correct for multiple testing within their statistical analysis. If they are deliberately not going to do that, then some justification for omission of this step must be provided.

**Response:** We thank the reviewer for this insightful and important observation. At this stage, we anticipate conducting a limited number of primary and secondary analyses, guided by our pre-specified outcomes (i.e., blood glucose regulation and satiety, with overweight and obesity as secondary outcomes). Given the exploratory nature of this systematic review and the expectation of a small number of pooled comparisons, we do not plan to implement formal corrections for multiple testing (e.g., Bonferroni).

However, we recognize the risk of Type I error inflation and will interpret all secondary analyses with appropriate caution, clearly distinguishing between confirmatory and exploratory findings. This decision is in line with recommendations from the Cochrane Handbook and recent literature that advise against automatic adjustment in the context of systematic reviews with limited and clinically heterogeneous data.

To clarify this point, we have added the following sentence to the "Statistical Analysis" subsection of the manuscript:

“Given the exploratory nature of this review and the limited number of pooled comparisons expected, we do not plan to apply formal corrections for multiple testing. All secondary analyses will be interpreted with caution and considered hypothesis-generating.”

We are grateful to the reviewer for prompting this clarification, which strengthens the transparency of our statistical approach.

*Best regards,*

*The authors.*
