# Supplementary material for: Consumption of sorghum-based products and their impact on blood glucose and satiety in adult patients: A protocol for systematic review and meta-analysis
Source: PLoS One. 2025 Sep 4;20(9):e0330998. doi: 10.1371/journal.pone.0330998 (PMC12410867; doi:10.1371/journal.pone.0330998)
Supplement: Attachment — Submitted filename: 12.06.25 Response to Reviewer Luis Track Changes.docx [file pone.0330998.s004.docx]

**Response to Reviewers**

PLOS ONE Decision: Revision required [PONE-D-25-15492]

Vitória, ES, Brazil, June 12^th^, 2025

Dear Federico Vita,

Academic Editor.

We sincerely thank the editors and reviewers for their valuable comments and suggestions regarding the structure and content of our manuscript. We have carefully addressed each point raised and provide a detailed, item-by-item description of the changes made below.

We are grateful for the thoughtful and constructive feedback, which has greatly enhanced the quality and clarity of our work. We fully agree with all the comments and have incorporated the recommended revisions throughout the manuscript.

Below, we provide detailed responses to each of the editor’s comments.

**Reviewer #1:** Thanks for your positive response to the comments. All of my points have been adequately addressed. Good luck with your study.

**Response:** We sincerely appreciate your positive feedback and thoughtful evaluation of our manuscript.

**Reviewer #3:** 1. Eligibility criteria: The authors have partially addressed my concerns regarding population inclusion and exclusion criteria. However, there is a inconsistency that must be resolved:

a. Major error in exclusion criteria: The revised manuscript (line 178) states that "adults (age > 18 years, of any ethnicity, diagnosed with diabetes mellitus or any other chronic disease)" will be excluded. This creates a fundamental contradiction with multiple elements of the study:
- The study rationale emphasizes sorghum's benefits for metabolic conditions
- The search strategy (Table 1) includes all adult patients without any exclusion
- The authors' response to reviewers states that adults with chronic conditions are included

b. Required resolution: The authors must either:
- Correct what appears to be a placement error and clarify that adults with chronic conditions will be included (consistent with their stated rationale and search strategy), OR
- If exclusion is intended, provide clear justification and revise the title, abstract, search strategy, and study rationale to reflect a healthy-adults-only population.

**Response:** We thank the reviewer for their careful reading and helpful comments.

We acknowledge the inconsistency noted in line 178 and confirm that this was indeed a typographical and placement error. Adults with chronic conditions such as diabetes mellitus **are intended to be included** in the study, as originally described in our rationale, search strategy (Table 1), and previous responses.

We have corrected the manuscript to reflect the accurate eligibility criteria, ensuring consistency across the rationale, methods, and inclusion/exclusion sections. The revised sentence now reads:
**“Exclusion criteria will children and adolescents, and adults with acute infectious conditions.”**

We appreciate the reviewer’s attention to this detail and the opportunity to clarify this important point.

2. Data Availability and Reproducibility: The authors adequately addressed my concerns by specifying use of the OSF repository, which is appropriately reflected in the manuscript.

**Response: OK. Thank you.**

3. Active Voice: While the authors incorporated active voice in line 133, this suggestion applies more broadly to improve manuscript readability. Multiple sections would benefit from active voice construction, particularly when describing researcher actions. Although passive voice serves appropriate functions (e.g., general statements or when the actor is unknown/insignificant), active voice typically enhances clarity in methodological descriptions. While not essential for approval, consistent voice improvement would enhance reader comprehension.

**Response:** We appreciate the reviewer’s thoughtful recommendation regarding the broader use of active voice to enhance readability. While we have incorporated some revisions in this direction, we understand that consistent application throughout the manuscript could improve clarity, especially in methodological descriptions.

If the editorial team deems it necessary, we are fully open to implementing these changes during the proof stage, in alignment with PLOS ONE’s final formatting and editorial guidance.

*Best regards,*

*The authors.*
